# Beliefs in Right Hemisphere Syndromes: From Denial to Distortion

**DOI:** 10.3390/brainsci15070694

**Published:** 2025-06-28

**Authors:** Karen G. Langer, Julien Bogousslavsky

**Affiliations:** 1 Department of Rehabilitation Medicine, NYU Grossman School of Medicine, NYU Langone Health, New York, NY 10016, USA; karen.langer@nyulangone.org; 2 Neurocenter, Swiss Medical Network, Clinique Valmont, Montreux, Switzerland

**Keywords:** anosognosia, awareness, denial, beliefs, right hemisphere

## Abstract

Striking belief distortions may accompany various disorders of awareness that are predominantly associated with right hemispheric cerebral dysfunction. Distortions may range on a continuum of pathological severity, from the unawareness of paralysis in anosognosia for hemiplegia, to a more startling disturbance in denial of paralysis where belief may starkly conflict with reality. The patients’ beliefs about their limitations typically represent attempts to make sense of limitations or to impart meaning to incongruous facts. These beliefs are often couched in recollections from past memories or previous experience, and are hard to modify even given new information. Various explanations of unawareness have been suggested, including sensory, cognitive, monitoring and feedback operations, feedforward mechanisms, disconnection theories, and hemispheric asymmetry hypotheses, along with psychological denial, to account for the curious lack of awareness in anosognosia and other awareness disorders. This paper addresses these varying explanations of the puzzling beliefs regarding hemiparesis in anosognosia. Furthermore, using the multi-dimensional nature of unawareness in anosognosia as a model, some startling belief distortions in other right-hemisphere associated clinical syndromes are also explored. Other neurobehavioral disturbances, though perhaps less common, reflect marked psychopathological distortions. Startling disorders of belief are notable in somatic illusions, non-recognition or delusional misattribution of limb ownership (asomatognosia, somatoparaphrenia), or delusional identity (Capgras syndrome) and misidentification phenomena. Difficulty in updating beliefs as a source of unawareness in anosognosia and other awareness disorders has been proposed. Processes of belief development are considered to be patterns of thought, memories, and experience, which coalesce in a sense of the bodily and personal self. A common consequence of such disorders seems to be an altered representation of the self, self-parts, or the external world. Astonishing nonveridical beliefs about the body, about space, or about the self, continue to invite exploration and to stimulate fascination.

## 1. Introduction

The mysteries behind unawareness of paralysis and other symptoms in cerebral disorders persist, inviting speculation as to the nature of belief behind the veil of ignorance. Anosognosia is one of those puzzling and unsolved enigmas of the right hemisphere.

Over 2000 years ago, Seneca wrote to Lucilius, depicting what we now know as cortical blindness (i.e., ‘Anton’s syndrome’), as Critchley [1] noted. Montaigne reported and translated the passage of Seneca, “This fool has suddenly lost her sight: I tell you a strange, but a very true thing: she is not sensible that she is blind, but eternally importunes her keeper to take her abroad, because she says the house is dark”… (Book II, Chap. 25, p. 482) [2].

In the late 19th century, a few rare case examples of unawareness were documented. Unawareness of blindness in cerebral dysfunction was reported by von Monakow in 1885 and by Anton in 1899, the latter describing a ‘seelenblindheit (psychic blindness)’ for the impairment (Anton’s syndrome) named after him (p. 173) [3]. Unawareness of paralysis was noted by Pick in 1898, and of left bodily unawareness with delusions by Zingerle in 1913 [see Papagno and Vallar] [3].

Nonetheless, anosognosia was one of the first clinical syndromes to receive wide attention as a disorder of awareness. First described by Joseph Babinski in 1914 [4], he originated the notion of a real unawareness syndrome with a precise definition, specifically of hemiplegia. Importantly for the syndrome, Babinski [4] wondered whether anosognosia for hemiplegia was a right hemisphere phenomenon. Throughout the decade to follow, Babinski and his colleagues were concerned with a motivated intentionality of non-acknowledgement of paralysis, versus a simpler ignorance or lack of knowledge. Over a hundred years later, this question still sparks animated discussion.

One of the first questions that Babinski addressed regarding the new phenomenon of anosognosia was whether it was real or not, ultimately concluding that it was neither feigned nor dissimulated. Does anosognosia result from a cognitive limitation so pronounced that the disability could not be recognized? Babinski addressed this point by restricting the cases to those who did not have dementia or profound cognitive loss. But, what about milder cognitive loss? And what of the possibility that unawareness is really a psychic attempt at self-protection from the knowledge of a reality too painful to acknowledge? These ideas and their theoretical underpinnings are discussed in detail below.

## 2. The Nature of the Problem: Belief in the Corridor of Unawareness

Beliefs are intrinsically linked with thinking and, hence, brain functioning [5]. Beliefs, and the failure or difficulty to update them, are suggested to play a pivotal role in the development of unawareness or anosognosia [6,7]. The fundamental nature of the problem concerns how patients can remain ignorant of, or unaware of, what is otherwise so obvious, and what is at the core of this (distorted) belief?

As a central aspect of human experience, belief adds an unseen layer to phenomenological interpretation. Anosognosia’s perplexing presentation demonstrates that the patient does not believe that s/he is paralyzed. Some patients may be simply unaware and ignorant of a disability (with a distorted view of their situation), others may display an active disbelief in paralysis. Babinski’s second patient specifically expressed the belief that she was not paralyzed [4] when stating explicitly “…It is not as if I am paralyzed” (p. 6) [8]. Despite eventual (explicit) verbal ‘admission’ of paralysis upon confrontation, one patient (implicitly) did not appear to believe it [9]. Beyond the passive ignorance of a disability, there is an explicit expression of disbelief [10], or indeed, a belief contrary to the facts of visible physical reality. In this vein, it is helpful to note, as Proust had prior remarked, that facts and beliefs may exist independently of each other [5].

It is important to recognize, in addition, that the unawareness and denial of paralysis or disability are themselves not identical, and may sometimes be dissociable. Double dissociation has been demonstrated [11], both in case examples of concurrent awareness of limitation or paralysis and failure to acknowledge it in interview, and in acknowledgement of paralysis with failure to appreciate functional performance consequences. Others have also reported that a patient might eventually acknowledge the paralysis but continue to deny functional consequences [12,13], so that awareness of paralysis is another matter from awareness of its consequences, as originally suggested by Critchley [1].

Clinicians have been startled, even disturbed, to witness the steadfast resolve with which patients stick to erroneous beliefs about the self and the body. Such right-hemisphere disorders of awareness and belief about the body and the self have been classified as body-image disturbances by Critchley [1] and others [14]. Some clinicians thought that, by demonstrating the paralysis to the patient, or by displaying failure of attempted movement, sufficient contrary evidence would convince the patients to alter their erroneous beliefs, in favor of concordance with reality. Seasoned observers were struck by the finding that this was not the case. The patients remained adamant that they were not paralyzed or expressed the conviction that they were moving the paralyzed limb [4], or could move it [15]. Rationalization attempts to explain a loss or deficit as a “little problem” (e.g., like a “recent cold”). The problem that is acknowledged is often minimized. As the patient is challenged, the rationalizations may become more entrenched and elaborate. Many rationalizations appear to be attempts to make sense of those limitations that are perceived (albeit without awareness of the paralysis), or to impart meaning to an incongruous set of facts. Some bear resemblance to a prior condition to which a patient may remain mentally mired. At other times, rationalizations appear devoid of such logic, sounding simply incredible. Time and time again, reluctance to relinquish such erroneous beliefs in the face of confrontation or challenge marks the conversations between examiner and patient.

The problem of reality-discordant belief is noteworthy, and one continues to wonder how a patient can maintain a belief that is so contradictory with the obvious facts of reality about hemiplegia, when other aspects of their reality-awareness appear preserved (i.e., patients can be aware of other deficits that are concurrent, or previously existing difficulties, such as phlebitis or back pain [4], or fatigue and a hernia [9]). Others interpret the deficit in a way that simply makes sense to them or suggests a plausible alternative explanation, such as a sprain or fractured arm [1].

Ultimately, it is remarkable though that the notably varied explanations for anosognosia (as a model for unawareness and belief distortions) each serve to explain some, but not all, the associated phenomena [5,16]. In light of that, and the fact that case exceptions can be readily found, it has been suggested to consider the possible concurrent operation of more than one source of unawareness [17], and it seems most “likely that several mechanisms play a synergistic role in most of these pathological conditions” (p. 131) [5].

In the following section we explore the explanations of the distortions in belief, using anosognosia both as a model and as the original syndrome of unawareness, the prototype from which explanations of disbelief in other disorders were developed.

## 3. Nonveridical Belief in Anosognosia

### 3.1. The Role of Sensory Losses

The original neurogenic account dating over 100 years ago centered on the key role played by sensory losses and their linkage to anosognosia [4] (though it was later suggested they do not fully explain it [18]), or the role of sensorimotor input plus proprioceptive signals and kinesthetic information regarding the limb [9]. This fundamental idea of an important contribution of such proprioceptive input is generally widely accepted, with occasional exception [11], and sensorimotor or proprioceptive information may be considered necessary though not altogether sufficient as a condition for unawareness of a paralyzed limb to develop [19].

### 3.2. Cognitive Contributions

Early clinical depictions of anosognosia and descriptions of the syndrome have withstood the test of time. Unlike directly detectable cognitive loss, such as in amnesia or aphasia, anosognosia presents with an erroneous belief against a background of otherwise relatively or grossly preserved intellect or cognition [4].

Babinski astutely excluded the confounding effects of general cognitive decline or dementia. In fact, Babinski omitted the cases presenting with marked cognitive decline from consideration. By restricting the cases as he did to those without obvious intellectual loss, he preserved the crisp clarity of a challenging new syndrome, adding to it heuristic value. The neurological context, historical background, and the clinical conceptualization of Babinski’s anosognosia has elsewhere been traced [10].

In the contemporary study of anosognosia, there is still some debate about the relative importance of cognitive decline in the development of unawareness. The interface between cognitive and sensory factors is especially of interest [20]. Addressing the sensory together with the cognitive, one novel theory has incorporated both, proposing that the discovery of sensory losses requires the use of inferential reasoning and self-observation so that cognitive difficulties may hinder the discovery of hemiplegia [19]. In this discovery theory, there is no fundamental perception of loss of sensation; instead, sensory loss “must be discovered by a process of self observation and inference” (p. 233) [19].

Overall, though, unawareness in anosognosia is not specific to any particular cognitive deficit, as there may be association with some, but not other, deficits [21]. Cognition has not generally been thought to be causal in anosognosia, as many patients have grossly preserved cognitive skills, though severe or generalized cognitive decline may play a role [22]. Further, there are studies that have demonstrated [23,24] that unawareness of deficit in anosognosia is a specific problem, separate from cognitive decline. The role of cognition in anosognosia is still deliberated [25,26]. Similarly, no specific cognitive or psychiatric cause has been found for the distortions in another disorder, the supernumerary phantom, and neurogenic causes are proposed.

### 3.3. Feedback, Hemispheric Disconnection, and Cerebral Asymmetry

Disturbances of a central conscious awareness system [27] or, alternatively, of modular modality-specific awareness systems [23] have been proposed. Various explanations for anosognosia focus on feedback and monitoring impairments to account for the puzzling features of unawareness; these are elsewhere compiled and reviewed [28]. Faulty feedback mechanisms may lead to the disturbed monitoring of reality, and diminished awareness. One prominent theory proposes a feedforward mechanism [29,30] invoked to explain the discordance between the intended (intentional) expectations and the perceived movement. Feedforward dysfunction is also suggested in the phantom third limb that Critchley described [1], where there exists an illusory belief in a third (nonparalyzed) arm, with discordance between the intention and the perceived movement [5,31]. By extension, self-awareness was suggested to depend on sensory information, attention to the body and the space where it is positioned, and body-representation informed continuously by (feedforward) expectations and feedback of the results [32]. These and other such monitoring theories are elsewhere reviewed [33]. Such theories generally propose a system for action which integrates “feedforward” signals, while comparing the intention of movement with the actual execution of movement. As a leading proponent of the feedforward hypothesis, Heilman [30] has suggested that there is no expectation of movement without the attempt to move the limb, and thus without moving the arm “there will be no discord that leads to discovery” (p. 30) [30]. In this vein, empirical support for the notion of faulty action monitoring involving feedforward signals has been provided, along with the suggestion that both defects in the processes of the motor comparator plus general monitoring defects can lead to unawareness in anosognosia [34]. Insufficient sensory feedback regarding limb position has also been posited, with inaccurate estimates of position sense suggested to be based not on the sensory feedback, but rather, the sequences of motor commands [35].

Furthermore, a shared anatomical association between motor awareness and motor control has been suggested [36]. Both denial and inaccurate belief of moving were explained by the investigators who suggested that denial was associated with lesions in areas mediating motor act programming, and that sparing of some premotor activity may lead to a “distorted representation of the intended motor act, which is responsible for the false belief of being able to move”(p. 490) [36].

A disconnection syndrome theory proposes disturbed interhemispheric communication [37,38]. Variants of this model include the novel idea that, when the left hemisphere becomes somewhat disconnected from the right hemisphere by cerebral injury, it offers its own explanations for observed losses, or that an asymmetry results. Importantly, it has been suggested that a left hemisphere unbridled of the influence of the corresponding right-hemisphere may generate explanations in the form of rationalizations, or denial, to account for new information regarding observed losses [24].

Such disconnection theories attempt to explain hemineglect, and other phenomena associated with cerebral dysfunction, in the context of disturbances of neuronal connectivity [39]. Notably, the two hemispheres possess two differing processing styles, with a ”more perceptive or emotional consciousness” of the right hemisphere and with the ”more analytical or linguistic processes of the left hemisphere” (p. 11) [20], whose disruptions may produce imbalances and lead to vast distortions of phenomenal interpretation.

### 3.4. Emotional Factors and Denial

Much debate has been sparked around the motivational basis of unawareness of disability, as opposed to a simpler lack of knowledge. The relative influence of ignorance and absence of knowledge, versus the adamant resistance or denial of unawareness, remains a question as relevant today as when first entertained a century ago.

Babinski [4] himself hinted at the possibility of an emotionally-protective effect (which is not identical with a motivated denial) to the unawareness, stating that the patients’ families considered the unawareness to be providential, protecting the patients, their loved ones, and resolutely asked to leave their loved ones undisturbed, with their serenity preserved. It was intimated that this protective aspect operated by masking awareness. The notion that there may be ‘bliss’ in unawareness, that a patient may be “blissfully unaware” [40], captures the idea of a potentially protective cushion. A century later, Lazarus evocatively suggested that “rather than equating the use of illusion with pathology, a more appropriate and interesting conclusion would be that mental health requires some self-deception” (p. 9) [41]. There is evidence both in favor and against the notion of such psychic armor.

Contemporary discussions underscore the complexities of anosognosia and the still-contested causes of denial and belief distortions. The more flagrant neurobehavioral response of denial has been noted in other neurological disorders, such as denial of memory loss in Korsakoff syndrome [42] and unawareness of language disturbance in Wernicke’s aphasia [43]. Nor is denial of illness restricted to cerebral or neurological disorders; extensive work on denial in medical illness has been elsewhere well-reviewed [44].

One study found that, in certain patients with serious illness (i.e., stroke, cardiac, lung cancer), “the effective use of denial protects the seriously ill patient from facing a reality characterized by frustration and despair” (p. 757) [45]. The protective effects of performance overestimation (i.e., less awareness) were reported in patients with stroke for quality of life and affect-related outcomes [46]. On the other hand, it is important to note that denial of illness or anosognosia immediately after acute stroke does not preclude the presence of depression [43,47]. Behavioral depression indicators were present in anosognosic patients [48], depression was found equally in patients with or without anosognosia [49], and anosognosia and depression may co-exist [50,51]. Patients who are unaware of their disability may yet develop sadness, even progressing to depression [51]. Acute behavioral denial in stroke patients was independent of anosognosia and associated with decreased subjective fear and increased occurrence of delayed depression [52]. Denial of illness by cardiac patients was deemed adaptive during acute recovery, but maladaptive later on (p. 109) [53]. Many have suggested that, at least early after onset or in recovery, some denial may be adaptive and protect the patient from being overwhelmed, but that later it may prove maladaptive [54], or impede functional rehabilitation progress (i.e., in patients with neglect plus anosognosia) [55]. The presence of denial in physical illness provides a context for viewing adaptive versus maladaptive uses, the latter of which may interfere with the development of more positive coping strategies [44]. These findings suggest a time-based dimensionality in the adaptative of use of denial. Finally, Lazarus [41] has offered a compelling framework for determining whether denial may be adaptive or maladaptive.

The notion of a motivated or emotionally driven denial has been among the most influential concepts concerning the sources of unawareness in anosognosia. One early idea proposed an “organic repression”, as introduced in works by Schilder, expounding his study of the body-image. Schilder’s view [56] is of a unity of the organic and the functional that encompasses individual personality plus sensory inputs which are constantly compared with prior experience. The repression is not psychological, per se, but rather could reflect an “instinctive urge to overlook” the paralyzed side (p. 28) [56]. Later, supplemented by the idea that there may be a “strong desire not to suffer the disability” (p. 135) [57], along with various neuropsychological defects, the notion of a motivational component to denial of disability was developed. Soon thereafter, starting in the 1950s, the thrust of the arguments for psychogenic causes took hold, proposing that unawareness reflected a motivated process of denial, both in its development and in its persistence. In a series of works on unawareness and denial, Weinstein and Kahn [58,59] studied patients with what was termed explicit denial and implicit forms of denial. It was argued persuasively that patients deny disability because of perfectionistic tendencies or intolerance of the defect, and because of perceived weakness, tendencies that reflected premorbid personality characteristics. The denial of illness was thought to carry symbolic meaning that could be deciphered, if not directly understood. Within the context of brain dysfunction, the expression of unawareness might vary from disavowed loss to underestimation of the limitation or overestimation of the ability. Anosognosia was explained as the unawareness of hemiplegia, which could be accompanied in certain patients by the blatant denial of hemiplegia. Throughout the decades that have followed, this view was popular in the neuropsychiatric literature, and an emotionally-based denial theory dominated the field for over three decades. Weinstein subsequently updated his view, adding that disrupted neural interconnections lead to anosognosia and diminished appreciation of emotional relevance, concurrently with the development of metaphoric “representations of an ’inner’ reality” (p. 255) [60]. Friedland and Weinstein [61] also later noted that the hemispheric asymmetry (i.e., the preponderance of right, rather than left, hemisphere involvement) in anosognosia could not be properly explained by purely motivational theories. Adding to the complexity is the existence of asymmetry, as noted in the role of the right hemisphere in emotional behavior [62] (see the reviews in [63,64,65,66,67]). Furthermore, anosognosia has recently been suggested to reflect the confluence of both cognitive (i.e., attentional and intentional impairments) and defensive denial [68] operations. Current perspectives on unawareness and motivated denial in the context of hemispheric asymmetries and emotions may be found in the reviews (see the discussions [68,69,70,71]).

The modern approach to the theory of a motivated denial considers the evidence in favor of a psychological defense, including implicit awareness, fluctuations over time, improvements with psychotherapeutic interventions, and the use of third-person discourse [69,72]. Rather than claiming that anosognosia is purely psychogenic in nature, some argue that any cognitive deficits related to cerebral dysfunction “that cause wishful emotions to undermine realistic cognition” (p. 22) [69] can be involved. The main argument against the psychological theory is the relative lack of the phenomenon in right hemiplegia. Indeed, one review noted that anosognosia is preponderantly associated with left hemiplegia (with reported frequencies (p. 294) ranging from 28% to 85% for right hemisphere stroke, versus 0% to 17% for left hemisphere stroke) [73]. Many of the other arguments against the motivated denial theory, including selectivity of denied deficit, recovery and time-dependent denial, as well as contrasting attitudes (such as denial versus obsessive focus), examples of which may be attitudes of misoplegia [74,75] or acute hemiconcern [76], among others, have been discussed in detail [77].

Clinically, denial as “an attempt to cope” albeit with a “partially recognized problem” has been distinguished from unawareness (of the self) as “a failure to recognize a need to cope” (p. 200) [78], and the complex challenges of distinction between anosognosia and denial continues to draw attention in the sphere of assessment [79,80]. Some suggest that anosognosia ought to be reserved [27] for the depiction of unawareness having an “organic” or neurogenic basis, distinguished (when possible) from motivated denial, a form of psychological defense mechanism. Others have decisively retained the term anosognosia in its classical sense, referring specifically to unawareness of hemiplegia [24]. Denial as a behavioral response involves adaptation or maladaptation to subjective distress [44], with anosognosia more classically defined as a dearth of knowledge with “an ignorance or fundamental unawareness of condition” (p. 443) [81]. Denial of paralysis has been differentiated from anosognosia not only quantitatively, as a matter of degree, but qualitatively. Indeed, anosognosia as “a mere deficiency in awareness, a poverty of insight or perhaps the operation of an organic repression” has been distinguished from “denial of the self-evident facts of paralysis” (pp. 233–234) [1], the latter reflecting more psychopathology. Empirically, anosognosia has been differentiated from behavioral denial, both as independent reactions [43]. The unbudging tenacity with which delusional beliefs are held has been described as obstinate denial [12], based upon Critchley’s [1] descriptions. More pathological manifestations of denial may require that the patient disregard and even dismiss the information received by the senses (p. 234) [1], reflecting the actively delusional nature of the problem.

Importantly, more contemporary views regarding neurogenic and psychogenic sources of unawareness suggest that, just as in the case of post-stroke depression, strict distinctions between the organic and the psychological may be somewhat artificial [82]. A multidimensional approach to another bodily awareness disorder, asomatognosia, proposes a broad perspective to understand the body schema disorders, considering neurological, cognitive, emotional, psychological, and contextual factors [26]. Lastly, the line of demarcation between these startling disorders is not fixed, and they may merge or alternate with one another (p. 225) [1], flowing into the fluid landscape of right hemisphere disorders.

## 4. Belief in Other Cerebral Disorders

### 4.1. Belief Distortions

Far from being uncommon in neurological disorders, unawareness or denial of corresponding impairment (e.g., unawareness of blindness noted in Anton syndrome in 1899 [83]), of disturbed language in Wernicke’s aphasia [84], and of memory loss in Korsakoff syndrome [42] has been recognized.

Delusional beliefs, though encountered perhaps less frequently than other, milder disturbances, are particularly confounding [25]. Delusional ideation in acute right hemisphere stroke has not been found to be related to prior psychiatric history [85], and preexisting diffuse atrophy may contribute to the clinical picture in delusional conditions [86]. One explanation for such delusional ideation hypothesizes that the left hemisphere may create narratives if there is limited right hemisphere information [87,88].

Belief distortions may be reflected strikingly in disorders of asomatognosia (i.e., the nonrecognition as one’s own of unilateral body parts or side, or the experience of their “nonexistence” (p. 912) [89]), the supernumerary phantom (i.e., the illusory subjective sense of a third arm or leg [1]), and somatoparaphrenia (i.e., the delusional denial of limb ownership plus the misattribution of belief, i.e., that the limb belongs to another person [89]), appearing as well in other attitudinal disturbances, such as misoplegia (attitude of contempt or hatred for a paralyzed limb with pathological features of verbal or physical aggression possible) [74,75]. Extrapersonal spatial neglect is marked by the belief that the “world-space” is full and veridical as experienced, and not as though there is a part of space that is absent to experience [90]. Similarly, the neurologically-based delusional beliefs in Capgras syndrome (belief that imposters have replaced significant others) and other misidentification syndromes are marked by the tenacious nonconformity with the facts of reality [85,91,92,93,94,95,96].

The rather astonishing manifestations of belief distortions and the accompanying phenomena of these predominantly right hemisphere syndromes are further reviewed [25]. A comprehensive glossary details these and similar disturbances (pp. 141–147) [97]. The progression of such disorders along a continuum of psychic severity [1] is apparent in the more dramatic of the delusional or confabulatory content. By extension, the advancement from unawareness to flagrant denial reflects a qualitative transformation into another realm of psychic disorder [1]. Misidentification syndromes are, similarly, examples of psychopathological belief distortions in cerebral dysfunction whose features may also present with a continuum of disturbance [98,99]. The more bizarre the beliefs or confabulatory the contentions of the patient, the more likely it will be to diagnose a psychotic-level dysfunction. Classified by Critchley as an “organic paranoid reaction” (p. 235) [1], the manifestations appear in the startling features of disorders such as somatoparaphrenia.

### 4.2. Distinctions and Parallels

The relationship between the disorders involving awareness in right hemisphere dysfunction is also germane. These conditions may be found in concert [1], particularly anosognosia and neglect [51], and associations have been suggested between anosognosia for hemiplegia and hemineglect [11,73,100,101], which is of heuristic and theoretical interest. Nonetheless, they are independent conditions, and dissociations have also been found between anosognosia for hemiplegia and neglect [23], between unawareness of illness and of neglect [22], between motor neglect and anosognosia [102,103], between awareness of motor deficits and (drawing) neglect [104], and between the patients’ expressed verbalization and intentional action [11]. Awareness of the symptoms of the neglect phenomena may be distinguishable and task-dependent [105]. Anosognosia may occur without neglect, with personal neglect found in about one third, and visuospatial neglect in about one half, of anosognosic patients [106]. Taken together, these and other findings have added support for the conclusion [107] that anosognosia for hemiplegia is not simply an effect or the secondary manifestation of related disorders of left spatial inattention or neglect. Moreover, anosognosia portrays a belief that the paralyzed limb is not paralyzed, while conversely, in motor neglect, patients behave as if they cannot move the limb despite movement possibility, with different unawareness content for anosognosia (i.e., for paralysis) and hemineglect (i.e., for contralesional half body or external space) [81].

Additionally, awareness of the spatial representation of the body and a sense of ownership of the body parts were likewise suggested to be different processes, in a comprehensive review [108], and these authors noted further that, in general, studies indicated independence of anosognosia from somatoparaphrenia. A disturbed sense of limb ownership was independent of anosognosia and personal neglect, suggesting they are distinct or unique conditions with differing lesion patterns [109]. Furthermore, anosognosia and anosodiaphoria, involving awareness or reaction to limb function, are doubly dissociated from unawareness of limb ownership (asomatognosia) [110].

It is also interesting to note the contrast between two opposite phenomena, “hysterical paralysis” (i.e., the perception or belief that a non-paralyzed limb is paralyzed, without a brain lesion) and anosognosia (i.e., the perception or belief that a paralyzed limb is not paralyzed, with a brain lesion). In hysterical paralysis, it could be said that the patients are not aware of the ability, and in anosognosia, it is the disability of which they are unaware [111]. Hysterical conversion symptoms were associated in one study with subcortical premotor circuits affecting sensorimotor function and voluntary motor activity, with the suggestion that these circuits may also be involved in unilateral motor neglect [112]. The significance of the phenomenon of hysteria, including hysterical paralysis and mental representation, and its historical development over time, is discussed in detail [113,114,115]. Lastly, a recently identified body image disorder associated with the right parietal lobe, apotemnophilia (sometimes also referred to as xenomelia), involves the wish for an amputation of a healthy limb, and also displays ”similarities with neurological symptoms (somatognosia, somatoparaphrenia, misoplegia)” (p. 19) [116].

While dissociable as separate disorders with unique characteristics, there is a unifying theme, characterized by Critchley [1], linking them under the heading of disorders of the body image, including anosognosia, neglect, anosodiaphoria, denial of paralysis with or without confabulation, asomatognosia, and phantom third limb [1]. Alternatively, the term body scheme has been used by several authors [14,117,118] for some of these disorders, depicting a powerful model of the time by which to characterize the nature of the various manifestations of right hemisphere dysfunction. These disorders and those other most dramatic manifestations of right hemisphere dysfunction are traced historically and illustrated clinically [25], as they paint the picture of a very active and colorful role for the “nondominant” right hemisphere [20]. The role of distributed networks has already been emphasized [20] for anosognosia and for neglect [105]. Other neural networks may include cortical and white matter with subcortical structure involvement in delusions of Capgras syndrome [96] and somatoparaphrenia [119]. Notably, disruptions of the neural networks that facilitate the assimilation or integration of the current with the past, involving perceptions, experiences, and memories, may interfere with the patient’s ability to make subtle adjustments for updating beliefs about the body and the self [5]. Right–left asymmetries resulting from neurological dysfunction have been implicated; the negative effects of a right hemisphere from impaired functions, together with a left hemisphere left “unchecked” to interpret and categorize, can lead to the delusional constructions of misidentification [87]. Delusions predominate with right hemisphere lesions, and are not easily redirected or corrected, in contrast with the less-lateralized confabulations, where patients may be redirected [87]. Importantly, it has been underscored that, when Capgras syndrome is diagnosed, a cerebral lesion should always be considered [91].

## 5. Implicit and Explicit (Un)Awareness

Paradoxically, on some (unspoken) level, patients may even appear to know about, or be aware of, the reality of disability (and of paralysis in anosognosia). Implicit awareness, or partial knowledge, despite explicit denial has been described in some anosognosic patients [16,27,72,77,120,121,122], and the possibilities of partial knowledge [11] and of “fractionability of consciousness” (p. 31) [77] have been proposed. Some patients may deny hemiplegia, yet avoid tasks requiring use of the paralyzed limb [77]. Others rather dramatically employed a strategy demonstrating an implicit awareness of impairment when asked to perform a motor task, despite an explicit denial of paralysis [123]. Furthermore, when asked to move the arm directly, some patients express the nonveridical belief that they have in fact moved, or could move, the limb [4]. Movement claims, even dissociated from explicit denial, were present in about one third to one half of the patients, for unilateral and bilateral movement attempts, respectively [123]. Dichotomies between nonverbal or implicit anosognosia and verbal explicit forms pose heuristic and clinical challenges [73], considering the factors of partial awareness, denial, coping, or a nonconscious processing system [124]. In neglect, a demonstration of ‘unconscious’ or implicit processing with various tasks and sensory modalities has suggested residual processing in areas spared by the lesion [100]. Importantly, evidence for the implicit processing of both perceptual features and semantic information in neglect has also been reviewed [105].

Not all patients, though, have shown demonstrable implicit awareness in anosognosia. In those patients with anosognosia lacking implicit awareness, a peculiar dichotomy has been observed, with explicit denial and delusional features despite generally preserved mental status, leading to the conclusion that the belief distortions are beyond explanation by simple cognitive issues alone [24]. Additionally, the curious finding of a patient who explicitly admits paralysis though seems not to ‘believe it’ raises the possibility, it is suggested, of bi-directionality in the dichotomy between implicit belief and explicit verbalization. As an illustration, a woman with left hemiplegia insisted that the fingers of her left hand would move if she simply tried, and asked the doctor whether he had seen them move [15]. As for the left lower extremity, she suggested the doctor step out of the way, so as not to be kicked when the leg moved. Later, she seemed to admit to left sided paralysis after repeated confrontation with her disability (“Since you say you think I’m paralyzed I believe I am”) (p. 96) [15], but very soon afterwards, she was asked if she believed this, and she said “No” (p. 96) [15]. Thus, beliefs may fluctuate, adding to the clinical picture of incongruity. Another patient became aware of paralysis, but reverted to the “false belief that she could move the arm” (p. 310) [125]. Similarly, after a second stroke resulting in left hemiplegia (the first with left homonymous hemianopsia), another patient (Olsen, cited in Nielsen) [126] displayed a comparable phenomenon. Denying that the affected limbs were hers, she explained that, although to her eyes they appeared to be her limbs, she felt as though they were not hers, and chose to believe her feelings rather than what she saw (Olsen’s case, cited in Nielsen) [126]. Another case of this somatic delusion of absence of an extremity persisted, and even though the patient recognized the logic of the evidence that the left hand was her own and was not missing, she still believed the hand did not belong to her [127]. Critchley [1] reviewed additional illustrative examples. A recent set of cases highlights the delusions or confabulations regarding the (predominantly left) paralyzed arm which characterizes somatoparaphrenia [128].

Keeping in mind this dichotomous uncoupling of implicit awareness and explicit denial, the issue of what constitutes actual belief is raised. One wonders whether the patient really believes that which is implicitly recognized, or rather, that which is explicitly denied. One may speculate that, at a “conscious” level of operation, that which is explicitly denied is believed, and is the basis of conscious action; whereas, at a more subconscious level of operation, the implicitly recognized content may be believed, forming the basis of reaction. In one early observation, some anosognosic patients were aware of the disability when queried in a third person format [11]. Furthermore, a lack of integration and dissociation between first- and third-person perspectives (egocentric, allocentric) in metacognitive beliefs has also been demonstrated empirically, such that the expressed belief is affected by the point of view used in the query in patients with anosognosia [129].

## 6. Body Representation

Right-hemisphere disorders of awareness and belief about the body and the self have been classified as body-image, utilizing Schilder’s [56] term, or body-scheme disturbances by Critchley [1] and Frederiks [14], and others. The famously important theoretical concepts of body representation and mental consciousness of the bodily-self, including schemata and the postural scheme [130] and ‘corporeal awareness’ (p. 545) [74], among others, took hold in a climate of nascent neurological curiosity and creativity [25]. A sophisticated nosology classifies disorders of the body schema based on factors including attitudes to the body plus defective (negative, absent, as in hemisomatognosia and personal hemineglect) versus productive (e.g., in delusions and illusions, such as somatoparaphrenia and supernumerary phantom) features [26].

As for the notion of the body image, an emphasis has been placed on the clinical exploration of the complex, multimodal subjective experience of the bodily self, along with an investigation of the still-uncertain sensorimotor mechanisms of neurologically-based disorders of body representation (e.g., asomatognosia, supernumerary phantom, and somatoparaphrenia, among others) [131]. Self-referential processing (both verbal and nonverbal) in disorders of the bodily and mental consciousness of the self is also considered in anosognosia [132]. Deficits with multi-sensory integration, along with proprioceptive deficits, may play a critical role in somatoparaphrenia [108], the latter another example of a productive manifestation in right hemisphere dysfunction [26,105]. Integration of the psychological, cognitive, and neuroanatomical factors in a holistic approach has also been proposed to account for somatoparaphrenia [128]. Interestingly, a comparison may be noted between somatoparaphrenia and the behavioral converse, that is, a disorder of pathological embodiment (e.g., alien hand syndrome). In such a disorder, the patient may have a sense of personal ownership of the foreign or alien hand and experience it as their own, with indications that it is incorporated into their mental somatosensory representation [133]. Pathologies of the bodily self may hint at, or shed light on, the underlying complex processes of the healthy bodily representation that still need to be discovered [26].

## 7. Updating Beliefs: Systems and Processes of Belief Development

A variety of explanations have been proposed, as noted above, to account for the unawareness and the range of belief distortions in anosognosia and other disturbances of awareness. A recent theory explains the development of delusions in terms of two factors, the first being a neuropsychological or other deficit, which explains the account of the unexpected event, and the second factor involving an impaired ability to reject a hypothesis on the basis of inconsistency or implausibility [134,135]. Still, one might further ask what putative process leads to difficulty in rejecting a hypothesis?

In anosognosia, patients come up with reasons that might credibly explain a paralysis with which they are confronted (typically in response to an observer’s query). This reason, as pointed out, is often one of an ailment or infirmity that either they experienced in the past, or experience concurrently. Beliefs may have their bases in memories of a prior state of health and wellness [136] or in their mental representations [5], and so they remain hard to modify and change.

As for belief maintenance, it has been suggested that there is relevance [5] to Ramachandran’s idea of left hemisphere predominance for applying consistency and stability in thinking, with right hemisphere specialization for monitoring and detecting large anomalies [24]. This theory proposes that the left hemisphere applies “consistency” even upon incoming anomalous data, but when there is too great a discrepancy between the existing idea and the anomalous data, the right hemisphere generates a shift in the paradigm [24]. The very process of a “change in perceptions when beliefs do not fit facts” (p. 131) [5] may be compromised by right hemisphere dysfunction, with the left hemisphere able to deny new facts of hemiplegia and retain prior, stable patterns of thought [5].

Beliefs, in this instance about the body and the self, involve “continual, interactive confrontation between actual perceptions and memories of past perceptions” (p. 132) [5]. Furthermore, beliefs necessarily fit in with existing notions, and information that does not correspond may be discounted. Thus, “‘New’ information that does not correspond to previous memories risks rejection because it challenges established experience and ways of thinking” (p. 130) [5].

The question of whether there exists a primary disruption to the (purported) systems involved in belief-updating has been addressed by several writers. One such hypothesis suggests that counterfactual beliefs are associated with lesions in the ventral attentional network and other associated, interconnected areas and that “self-awareness extends beyond local, retrospective monitoring … requiring also … beliefs about the self that go beyond the ‘here-and-now’ of sensorimotor experience” (p. 1) [6]. Deficits in integration of interoceptive and motivational signals due to striatal and insular lesions has also been suggested, resulting in difficulties personalizing “new sensorimotor information, and an abnormal adherence to premorbid beliefs about the body” (pp. 127–128) [137]. A novel study investigated the responses to a target word task; the investigators found that anosognosic patients (with adequate cognition implied) held onto their incorrect responses despite incongruity with new clues [7]. They concluded that a deficit in the “generation and adjustment of beliefs” may further anosognosia “when associated with concomitant losses in motor, proprioceptive, and/or attentional functions” (p. 1771) [7]. It is also suggested that “difficulties in affectively personalising new sensorimotor information … coupled with … adherence to past expectations of how the affected body parts should feel” results in the incorrect beliefs (p. 505) [138]. As for the delusional misattribution of limb or side to others in somatoparaphrenia, it has been suggested that there is a defect in the update of a dynamic body representation [139]. One unifying thread in these various hypotheses is the suggested difficulty updating the beliefs. The difficulty that patients encounter in updating their beliefs about their bodily self, or the factors that contribute to their adherence to outdated expectations despite new information, is a subject of significant interest.

One hundred years ago, Barkman [136] proposed that patients, when lacking sensory information about the left side, would revert to the past memory, that recalled the state of being well. That notion is still highly relevant today. Our contemporary view is that beliefs, difficult to change as they are, are rooted in the mental representations of memory of the body and the self [5]. Beliefs, established as inner and outer perceptions of the senses are integrated, may carry some emotional valence [5]. When new information does not “correspond to previous memories” (p. 130), it is not easily accepted because it does not conform with “established experience and ways of thinking” (p. 130) [5]. In other words, beliefs are part of the fabric of pre-existing thought patterns. There is no specific area in the brain which “can be considered as a privileged ‘belief centre’.” (p. 131) [5], and no localization is identified with belief development and maintenance. Although there is ample clinical evidence and metanalytic support [140] for the largely lateralized association with right hemisphere dysfunction, the disorders of awareness, limb ownership, and identity or misidentification involve notions of functional interconnectivity and neuronal networks [5,25,39,108,131]. Self-awareness and beliefs about the self, the body, and identity evolve through ongoing comparison with past experiences and thought patterns [5].

## 8. Summary and Conclusions

Interactions between awareness, emotion, memory, thinking, and perception, along with neuronal network interconnections, may play a crucial role in belief construction, maintenance, and updating [25]. Such processes involve established ways of thinking, prior experience, and previous memories [5], providing the framework for comparison and assimilation. The “continual, interactive confrontation between actual perceptions and memories of past perceptions” (p. 132) permits memories of the past to be subtly adjusted, as new perceptions, experiences, and expectations are integrated within the awareness of the body and the self [5]. In the case of neurological disorders of awareness, however, existing patterns of past experience from which new information differs, in the context of neuronal network disruptions, may eclipse simple belief modification. Beliefs and their puzzling distortions represent integral facets of predominantly right hemispheric cerebral disorders of awareness and neurobehavioral dysfunction, meriting innovative study and imaginative exploration.

## Data Availability

No new data were created or analyzed in this study.
